# Early Interim Chemotherapy Response Evaluation by F-18 FDG PET/CT in Diffuse Large B Cell Lymphoma

**DOI:** 10.3390/diagnostics10121002

**Published:** 2020-11-24

**Authors:** Hye Lim Park, Eun Ji Han, Joo Hyun O, Byung-Ock Choi, Gyeongsin Park, Seung-Eun Jung, Seung-Ah Yahng, Ki-Seong Eom, Seok-Goo Cho, on behalf of Catholic University Lymphoma Group

**Affiliations:** 1 Division of Nuclear Medicine, Department of Radiology, Eunpyeong St. Mary’s Hospital, College of Medicine, The Catholic University of Korea, Seoul 06591, Korea; prhlim@gmail.com; 2 Division of Nuclear Medicine, Department of Radiology, Yeouido St. Mary’s Hospital, College of Medicine, The Catholic University of Korea, Seoul 06591, Korea; iwao@catholic.ac.kr; 3 Division of Nuclear Medicine, Department of Radiology, Seoul St. Mary’s Hospital, College of Medicine, The Catholic University of Korea, Seoul 06591, Korea; 4 Department of Radiation Oncology, Seoul St. Mary’s Hospital, College of Medicine, The Catholic University of Korea, Seoul 06591, Korea; choibo67@catholic.ac.kr; 5 Department of Hospital Pathology, Seoul St. Mary’s Hospital, College of Medicine, The Catholic University of Korea, Seoul 06591, Korea; gspark@catholic.ac.kr; 6 Department of Radiology, Eunpyeong St. Mary’s Hospital, College of Medicine, The Catholic University of Korea, Seoul 06591, Korea; sejung@catholic.ac.kr; 7 Department of Hematology, Incheon St. Mary’s Hospital, College of Medicine, The Catholic University of Korea, Seoul 06591, Korea; saymd@catholic.ac.kr; 8 Department of Hematology, Seoul St. Mary’s Hospital, College of Medicine, The Catholic University of Korea, Seoul 06591, Korea; dreom@catholic.ac.kr (K.-S.E.); chosg@catholic.ac.kr (S.-G.C.)

**Keywords:** diffuse large B cell lymphoma, F-18 FDG PET/CT, interim response, response evaluation

## Abstract

Fluorine-18 fluorodeoxyglucose (FDG) positron emission tomography (PET)/computed tomography (CT) after one cycle of standard chemotherapy in patients with diffuse large B cell lymphoma (DLBCL) was assessed. Prospectively enrolled 51 patients had four PET/CT studies using the same protocol and system: at baseline and after one, three, and six cycles of chemotherapy (PET0, PET1, PET3, PET6). The PET1 and PET6 Deauville five-point score (D5PS) agreed in 60.8%, while PET3 and PET6 D5PS agreed in 90.2%. The absolute and percent changes of peak standard uptake value corrected for lean body mass (SULpeak) compared to baseline were significantly different between PET1 and PET3 (*p* = 0.001, *p* < 0.001) and PET1 and PET6 (*p* = 0.002, *p* = 0.001), but not between PET3 and PET6 (*p* = 0.276, *p* = 0.181). The absolute SULpeak from PET1 predicted treatment failure with accuracy of 78.4% (area under the curve 0.73, *p* = 0.023). D5PS, SULpeak, and metabolic tumor volume (MTV) were not statistically different between responders versus non-responders, or the one year disease-free versus relapse groups. D5PS and PERCIST responses showed 100% agreement at end-of-therapy. In conclusion, the responses after three and six cycles of therapy showed high degree of agreement. D5PS or MTV after one cycle of chemotherapy could not predict response or one-year disease-free status, but the SULpeak from PET1 was associated with response to first line therapy in DLBCL. Deauville and PERCIST criteria show high concordance.

## 1. Introduction

Diffuse large B cell lymphoma (DLBCL) is the most common non-Hodgkin’s lymphoma (NHL), approximately over 30% of all lymphoma cases, and shows aggressive clinical course. In Korea, incidence of DLBCL was 29.8% among all lymphoid malignancy from 1999 to 2012 [1]. Initial treatment of choice for DLBCL is rituximab, cyclophosphamide, hydroxydaunomycin, vincristine (Oncovin^®^), prednisone (R-CHOP) regimen. Remission following first line chemotherapy using 6–8 cycles of R-CHOP is expected in 50–70% of patients [2]. However, over one-third of patients need salvage treatment for refractory disease [3]. These non-responders suffer the toxic side effects of chemotherapy without clinical benefit, whereas an early change in treatment strategy may have been more helpful.

F-18 fluorodeoxyglucose (FDG) positron emission tomography (PET)/computed tomography (CT) has emerged as an important tool for the evaluation of Hodgkin lymphoma (HL) and aggressive NHL [4]. Most of the guidelines for DLBCL recommends FDG PET/CT scan at the time of staging and at end of therapy. However, interim FDG PET/CT scans during chemotherapy are carefully recommended because of the possibility of false positive results [5,6]. FDG PET/CT images can be assessed visually or by using semi-quantitative parameters. In FDG-avid lymphomas, visual analysis using Deauville five-point score (D5PS) is recommended in the Lugano response criteria and NCCN guidelines [4,7]. It is a simple tool based on visual grading of FDG uptake in the tumor compared to the liver, and does not apply different thresholds for interim or end-of-therapy assessment. Response criteria by International Working Group (IWC + PET) used visual assessment compared to background or reference organ to decide whether a PET scan is positive or negative [4,8]. Although this binary categorization has the advantage of easy clinical application, partial response could not be determined by PET findings alone. PET Response Criteria in Solid Tumors (PERCIST) 1.0 provides guideline for quantitative response assessment, but the criteria were developed for solid tumors [9].

NCCN guideline recommends D5PS-based interim FDG PET/CT response assessment after 2–4 cycles of R-CHOP in patients with DLBCL, but the exact time point for interim response varies across institutions [7]. If early interim response evaluation can predict the end-of-therapy response, clinicians can alter the planned treatment strategy accordingly and the patients can receive the more effective treatment. Theoretically, inflammatory reaction would be minimal early into chemotherapy, and early interim FDG PET/CT could perform better than the routinely used mid-therapy response, and the true-negative scan early into chemotherapy could represent remission [10,11]. It had been observed in solid tumors that early declines in FDG uptake could be seen in responders [12,13]. We wanted to further explore the tumor kinetics to R-CHOP therapy so to possibly guide personalized dose reduction or escalation decisions in the future. The primary aim of this study was to evaluate whether early response assessment by FDG PET/CT after one cycle of R-CHOP could predict the success of therapy and the patient’s disease-free status for the first year following therapy. We also wanted to compare the quantitative and qualitative response assessments at different time points.

## 2. Materials and Methods

### 2.1. Patients

Fifty-four consecutive treatment naïve patients with newly diagnosed DLBCL were prospectively enrolled from March 2012 to December 2017 at single tertiary referral center specializing in hemato-oncologic diseases. Inclusion criteria were pathologically confirmed CD20 positive DLBCL without prior lymphoma treatment, and age 19 years or older. Exclusion criteria were pregnancy and primary central nerve system lymphoma. All patients received standard R-CHOP as first line therapy.

This study was performed in accordance with the regulations of our hospital’s Institutional Review Board, which approved the prospective design (KC11EISI0293). All patients signed a written informed consent before study procedures. The study was registered in the ClinicalTrials.gov database (NCT01357733) on 23 May 2011 (https://clinicaltrials.gov/ct2/show/NCT01357733).

### 2.2. FDG PET/CT Acquisition

FDG PET/CT was performed 4 times using same imaging protocol and the same PET/CT system: at baseline (PET0), after 1 cycle (PET1), after 3 cycles (PET3), and after completion of 6 cycles of chemotherapy (PET6). PET1 was done as close to the beginning of second cycle as possible—2 to 48 h before the second cycle of R-CHOP was administered. The findings of PET1 were not used to change the course of therapy.

All patients fasted for at least 6 h before the FDG PET/CT scan. 5.2–7.8 MBq/kg of FDG was injected intravenously, and scanning began strictly 60 min later, allowing for variation of 5 min. Images were acquired using a combined PET/CT in-line system, the Biograph Truepoint (Siemens Medical Solutions, Knoxville, TN, USA). All patients were in the supine position during scanning. Non-contrast enhanced CT began at the vertex and progressed to the proximal thigh (120 kVp, 80 mAs, 3-mm slice thickness). PET scans of the same body region followed immediately. The acquisition time was 5 min per bed position, and 5–8 beds were obtained. The CT data were used for attenuation correction, and images were reconstructed using a standard ordered-subset expectation maximization algorithm (OSEM; two iterations, eight subsets). The axial spatial intrinsic resolution of the system was 4.2 mm at the center of the field of view.

### 2.3. Image Analysis, Response Evaluation, and Disease Status at 1 Year

All FDG PET/CT scans were independently analyzed by two experienced nuclear medicine physicians (HLP and EJH), both with over 10 years of experience in reading PET/CT images of lymphoma, using XD3 (Mirada Medical, Oxford, UK).

Response was assessed visually by D5PS, and patients with scores 1, 2, and 3 were considered to have achieved complete response. Scores 4 and 5 with reduced FDG uptake compared to baseline were considered partial response. Scores 4 and 5 with similar tumor burden were considered stable disease. Scores 4 and 5 with increased FDG uptake or development of new lesion were considered progressive disease. When the two readers designated a different D5PS, consensus was reached after discussion (HLP, EJH, JHO). Additionally, we classified the patients as early and late responders: early responder meant D5PS 1, 2, or 3 at both PET1 and PET6; and late responder meant the patient had D5PS 4 or 5 at PET1, but later had D5PS of 1, 2, or 3 at PET6. Patients with D5PS 1, 2, or 3 at end-of-therapy were considered to be responders to the first line therapy.

For quantitative analysis, we measured the peak standardized uptake value corrected for lean body mass (SULpeak) as described in PERCIST 1.0; the average activity within a 1 cm^3^-volume spherical region of interest centering on the hottest point in the tumor foci. The absolute and percent changes of SULpeak (absΔSUL and %ΔSUL) at early interim between PET0 and PET1 (PET_∆0–1_), at mid-therapy between PET0 and PET3 (PET_∆0–3_), and at end-of-therapy between PET0 and PET6 (PET_∆0–6_) were calculated. The %ΔSUL were calculated as 100 × ((SULpeak of PET0 − SULpeak of PET1, PET3 or PET6)/SULpeak of PET0). Per PERCIST 1.0, complete metabolic response (CMR) means the FDG uptake of lymphoma lesions were indistinguishable from that of the background; %ΔSUL ≥ 30% was considered partial metabolic response (PMR); %ΔSUL within 30% considered stable metabolic disease (SMD); and an increase in SULpeak ≥ 30% or appearance of new lesion was considered progressive metabolic disease (PMD). CMR and PMR were considered to represent responders; and the patients with SMD and PMD were considered as non-responders [14]. We also measured the metabolic tumor volume (MTV) of all lymphoma lesions. 1.5 × SULmean + 2 standard deviation (SD) of liver was used as the threshold to define the MTV. The absolute and percent change of MTV (absΔMTV and %ΔMTV) at PET_∆0–1_, PET_∆0–3_, and PET_∆0–6_ were also calculated. The SULpeak and MTV were measured independently by two readers and double checked for accuracy, with the measurements repeated when the numbers were not equal.

The FDG PET/CT findings were assessed for prognostic value for short term clinical outcome following completion of R-CHOP regimen. Patients who achieved complete metabolic response with R-CHOP and continued to have no image or histologic evidence of lymphoma recurrence during the first year of surveillance were considered to be disease-free at 1 year. Surveillance included physical examination and laboratory studies every 3 months, and contrast enhanced CT of the neck, chest, and abdomen at 6 and 12 months following the end of R-CHOP therapy.

### 2.4. Statistical Analysis

Statistical analysis was carried out using the Statistical Package for Social Sciences (SPSS) software (version 24.0). All quantitative values are expressed as mean ± standard deviation (SD). McNemar’s test was done for the agreements among the categorical responses and the paired t-test was performed to compare the SUL and MTV changes for PET_∆0–1_, PET_∆0–3_ and PET_∆0–6_. Differences between early and late responders were tested with Chi-square or Fisher’s test and t-test. Independent *t*-test for continuous values and Chi-square test for categorical values were performed to compare PET/CT findings between disease group and disease-free group at 1 year. Receiver-operating characteristic (ROC) analysis evaluated the prognostic value of the quantitative parameters for disease-free status at 1 year. The *p* values < 0.05 were considered to indicate statistical significance.

## 3. Results

### 3.1. Baseline Patient Characteristics

Of the 54 enrolled patients, one patient was excluded because the therapeutic regimen changed midway with progression after three cycles of chemotherapy, and two patients chose to drop out before completion of chemotherapy. In the end, 51 patients had complete set of four PET/CT studies, and the patient characteristics are shown in Table 1.

### 3.2. Visual Analysis of FDG PET/CT

**Figure 1:**
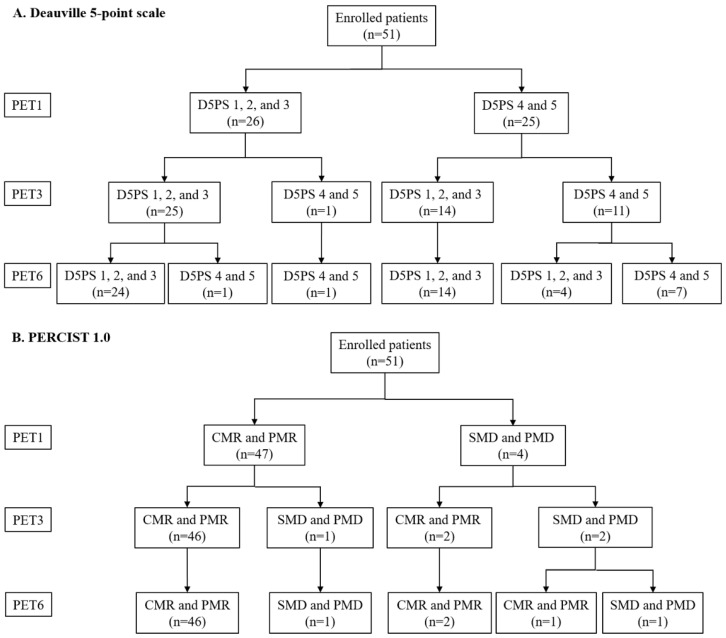
Number of patients according to D5PS (**A**) and PERCIST1.0 response (**B**) at different time points during R-CHOP chemotherapy: early-interim after 1 cycles of chemotherapy (PET1), mid-therapy after 3 cycles of chemotherapy (PET3), and end-of-therapy after 6 cycles of chemotherapy (PET6). Deauville scores of 1, 2 and 3 were grouped to represent complete response (**A**). According to PERCIST1.0, patients showing %ΔSUL ≥ 30% were considered responders; and those with %ΔSUL less than 30% or increase in SULpeak or appearance of new lesion were considered non-responders (**B**).

Patient flow charts according to D5PS and PERCIST 1.0 results are shown in Figure 1. The D5PS between PET1 and PET3 agreed in 70.6% (36 out of 51) of patients, and response between PET1 and PET6 agreed in 60.8% (31 out of 51) of patients. D5PS between PET3 and PET6 agreed in 90.2% (46 out of 51). There was no statistical difference in D5PS between PET3 and PET6 (*p* = 0.375).

We also classified the patients as early-responders and late-responders by the results of PET1 and PET6. Early-responders (*n* = 24) achieved D5PS 1, 2, or 3 at both PET1 and PET6. Late-responders (*n* = 18) had D5PS of 4 or 5 at PET1, but scores of 1, 2, or 3 at PET6. There was no significant difference in clinical characteristics (Table 2).

### 3.3. Quantitative Analysis of FDG PET/CT

Mean ± SD (range) of SULpeak at PET0, PET1, PET3 and PET6 were 13.3 ± 5.9 (2.3–27.1), 2.9 ± 2.5 (0.5–16.3), 2.1 ± 1.8 (0.4–10.1), and 1.8 ± 1.5 (0.4–10.7), respectively. The mean ΔSUL and ΔMTV are shown in Table 3. The %ΔSUL in detail at each time point for each patient were shown in Figure S1. The mean absΔSUL and %ΔSUL were different between PET_∆0–1_ and PET_∆0–3_ (*p* = 0.001 and *p* < 0.001, respectively), and between PET_∆0–1_ and PET_∆0–6_ (*p* = 0.002 and *p* = 0.001, respectively). However, the absΔSUL and %ΔSUL were not different between PET_∆0–3_ and PET_∆0–6_ (*p* = 0.276 and *p* = 0.181). Also, the mean absΔMTV between PET_∆0–3_ and PET_∆0–6_ was not statistically different (*p* = 0.127).

**Figure 2:**
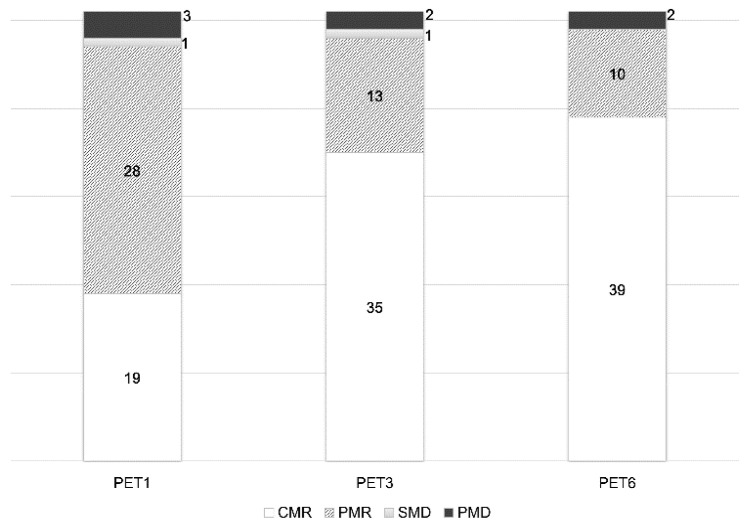
Number of cases at different time points for metabolic response categories according to PERCIST 1.0.

According to PERCIST 1.0, there were 19 CMR, 28 PMR, one SMD and three PMD cases seen on PET1, and 35 CMR, 13 PMR, one SMD, and two PMD on PET3 (Figure 2). At the end of therapy on PET6, there were 39 CMR, 10 PMR and two PMD cases. There was no SMD case at PET6. PERCIST 1.0 showed good agreement in categorizing the patients as responders or non-responders between PET1 and PET3 (94%, 48 out of 51), PET1 and PET6 (92.2%, 47 out of 51), and PET3 and PET6 (98.0%, 50 out of 51). There was no statistical difference in response categorization among PET1, PET3, and PET6 (PET1 vs. PET3, *p* = 1.0; PET1 vs. PET6, *p* = 0.625; PET3 vs. PET6, *p* = 1.0).

### 3.4. Comparison between Quantitative and Visual Analysis

**Figure 3:**
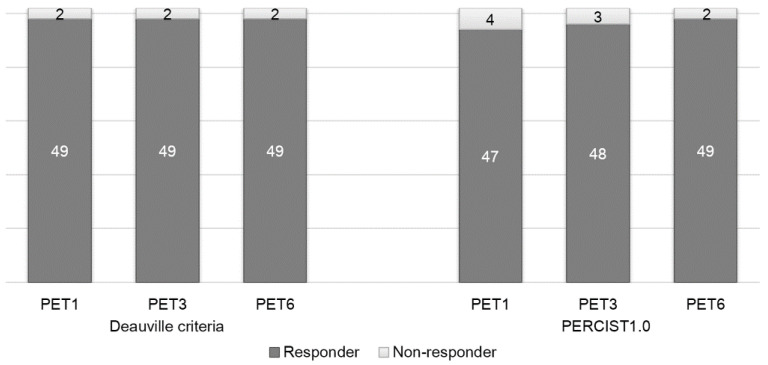
Number of responders and non-responders at each time point according to the two response criteria.

The number of responders and non-responders according to Deauville criteria and PERCIST1.0 are shown in Figure 3. On PET1, 23 of the 25 patients with D5PS of 4 or 5 could be classified as partial response as the FDG uptake was reduced. On PET3, 10 patients with D5PS of 4 or 5 showed partial response. On PET6, seven patients with D5PS 4 or 5 showed partial response. Response categorization according to the two evaluation methods was not statistically different through all three time points (*p* = 0.5 at PET1, *p* = 1.0 at PET3, and *p* = 1.0 at PET6). The qualitative and quantitative assessments yielded the same responses in 96.1% (49 out of 51) cases at PET1, 98.0% (50 out of 51) at PET 3 and 100% (51 out of 51) at PET6. The %ΔSUL at each time point according to D5PS are shown in Table S1.

### 3.5. Treatment Outcome and 1 Year Clinical Outcome

From the ROC analysis for predicting the end-of-therapy response, SULpeak measured from PET1 yielded area under the curve (AUC) of 0.729 using the cut-off value of 3.4, with overall accuracy of 78.4% (*p* = 0.023). The specificity and negative predictive value for predicting treatment failure were 81.0% and 91.9%, respectively. The sensitivity and positive predictive values were lower at 66.7% and 42.9%, respectively. The MTV from PET1 did not show significant accuracy for predicting treatment outcome with cut-off value of 3.1 cm^3^ (AUC 0.612, *p* = 0.292). The SULpeak and MTV from PET3 had meaningful AUCs (0.868, *p* < 0.001 and 0.770, *p* = 0.003, respectively), but the calculated cut-off values were low at 2.0 for SULpeak and 0.2 cm^3^ for MTV.

Of the 51 patients, there were 9 (18%) who experienced relapse or disease progression in the one-year period after completion of R-CHOP therapy. The remaining 42 patients (82%) remained disease-free. At all time points, the SULpeak and MTV were not different between the disease-free and disease present groups (Table S2). The absolute and percent changes in SULpeak and MTV compared to baseline were also not different between the disease-free and disease present groups (Table S3).

## 4. Discussion

This study was designed to perform early interim response evaluation after one cycle of R-CHOP, mid-therapy response evaluation after three cycles of R-CHOP and lastly end-of-therapy response under strictly controlled uniform imaging conditions. We were interested in PET response after one cycle to better understand the early kinetics of DLBCL tumor in relation to standard therapy [10]. Initial treatment of choice of DLBCL is R-CHOP immunochemotherapy regimen. However, 30–40% of patients with DLBCL fail to be completely cured [15]. There is a clinical need to identify the patients with DLBCL who will not reach complete remission from R-CHOP, and plan the treatment accordingly. Being able to identify early responders accurately could also open the way for dose reduction in the future. In our study, using cutoff value of 3.4 on FDG PET/CT after one cycle of chemotherapy, the SULpeak could predict failure to R-CHOP therapy (*p* = 0.023). However, the area under the curve of 0.73 would not be high enough for clinical application in individual patients.

There are two widely used response assessment methods using FDG PET/CT. In FDG-avid lymphomas, visual analysis using D5PS is recommended in the Lugano response criteria and NCCN guidelines [4,7]. In solid tumors, PERCIST 1.0 provides guideline for quantitative response assessment [16]. In this study, the qualitative and quantitative assessments yielded the same responses in 96.1% of the cases after one cycle of chemotherapy, 98.0% after three cycles, and 100% at end-of therapy. Although visual analysis using D5PS is relatively simple and does not require any time-consuming measurements, quantitative PET parameters may better express the continuum of patient’s response than the D5PS. In addition, there remains an issue of inter and intra-reader variability with D5PS [17,18]. In this study, PERCIST response categorization was steady over time with no statistical difference in response categorization among PET1, PET3, and PET6.

The role of interim FDG PET/CT in HL is clear and PET parameters showed high prognostic value [19]. In HL, interim FDG PET/CT is recommended [20] and D5PS is used to guide treatment planning after chemotherapy. However, the role of interim FDG PET/CT in DLBCL is still not fully established. For stages III and IV DLBCL, interim FDG PET/CT is recommended after 2–4 cycles of chemotherapy to detect non-responders. For stages I and II, FDG PET/CT is recommended for planning the radiation treatment field [7]. In order for interim FDG PET/CT to have a clear clinical role, the interim FDG PET/CT results should correlate well with the clinical outcome. Previous studies reported utility of interim FDG PET/CT, but included varying treatment regimens, times for interim FDG PET/CT, and different criteria for response evaluation [21,22,23]. The predictive value of interim PET on progression free survival also showed wide range of hazard ratios and positive predictive values [24].

One retrospective study compared the predictive value of FDG PET/CT parameters after 1 cycle of chemotherapy and two cycles of chemotherapy [25]. FDG PET/CT using percent change of maximum standardized uptake value (SUV) after one cycle of chemotherapy showed similar predictive value with the change after two cycles of chemotherapy. Because the endpoint of this paper was earlier than ours, it is difficult to compare the results directly. In a retrospective study, FDG PET performed after one cycle of therapy could predict outcome in DLBCL (*n* = 24) and classic HL (*n* = 23) [26]. In a different study, the SUV measurements from PET after two cycles of chemotherapy could predict event free survival with ROC area under the curve of 0.67, and overall survival with AUC of 0.74 [27]. However, in a more recent Nordic/US intergroup study with 112 DLBCL patients, FDG PET/CT after one cycle of chemotherapy could not discriminate patients with different prognoses [28]. There was one patient in this study with new lesions on FDG PET/CT after one cycle of chemotherapy. The new FDG avid nodes were in the axilla and mediastinum. This patient later had remission on PET3 and PET6. Such case suggests it would be premature to change the therapeutic plan based on PET1 findings.

Recently, several studies reported the predictive and prognostic value of FDG PET derived MTV in varying subtypes of HL, mantle cell lymphoma, central nervous system lymphoma, and Burkitt lymphoma [21,29,30,31,32,33]. In a study of 39 patients with NHL, the MTV from baseline FDG PET/CT was used for prognostication following stem cell transplantation [34]. According to our MTV data, the range of percent change in MTV after 1 cycle was from 17.9% to 100%, showing that in a few patients more than one third of the tumor burden remained. After three and six cycles, all the patients showed volume reduction of at least 81% and 87.5%, respectively. The reduction of MTV after one cycle of chemotherapy was significantly less that the reduction after three and six cycles of chemotherapy. However, the lower absolute and percent MTV changes after one cycle did not have predictive value for detecting poor response to R-CHOP or relapse within one year.

Previous studies also reported that molecular subtypes of DLBCL affect prognosis [35,36]. In our study population, 20 patients were germinal center B cell (GCB) like subtype and 25 patients were non-GCB subtypes. The remaining six patients had unspecified molecular subtypes. On PET6, there were nine of D5PS 4 or 5, and six were non-GCB subtype, one was GCB, and two were unclassified.

The limitations of this study were the relatively small number of patients with single center experience, and that a relatively short-term clinical outcome was assessed. However, in this prospective study, all the enrolled patients received the same protocol of R-CHOP and were imaged using the same PET/CT scanner following strict imaging conditions. We also plan on performing long term prognostic analysis. Larger multicenter studies that assess the quantitative parameters such as MTV are also warranted to establish their prognostic value in patients with DLBCL.

The patients were at liberty to view their FDG PET/CT images whenever they requested. The FDG PET/CT images, especially the maximum intensity projection (MIP) images, allowed the patients to intuitively grasp their tumor burdens. Though it was clearly explained that early response does not represent the end-of-therapy response, responding early interim PET images had the positive role of encouraging the patients to continue their long chemotherapy courses.

## 5. Conclusions

Early interim response assessment of therapy after one cycle of R-CHOP by both qualitative and quantitative FDG PET/CT parameters was different from the end-of-therapy assessment. Responses after three cycles and six cycles of therapy showed high degree of agreement. D5PS or MTV after one cycle of therapy could not predict response to chemotherapy or one-year disease-free status, but higher SULpeak from PET1 was associated with failure to achieving complete remission with first line therapy in DLBCL. The visual Deauville scale and quantitative PERCIST criteria resulted in identical response categorization at end-of-therapy.

## Figures and Tables

**Table 1:** Patient demographics (*n* = 51).

Variable	Number (%)
Age	
Mean ± SD ^a^ (range)	54.6 ± 14.5 years (21–81)
≤60	30 (58.8)
>60	21(41.2)
Sex	
male	31 (60.8)
female	20 (39.2)
Ann Arbor stage	
I–II	21 (41.2)
III–IV	30 (58.8)
LDH ^b^	
normal	25 (49.0)
elevated	26 (51.0)
ECOG ^c^ Performance status	
0–1	40 (78.4)
2–4	11 (21.6)
International Prognostic Index	
0–1	17 (33.3)
2	10 (19.6)
3	13 (25.5)
4–5	11 (21.6)
Number of extranodal site	
0–1	22 (43.1)
≥2	29 (56.9)
Subtype	
GCB ^d^	20 (39.2)
Non-GCB	25 (49.0)
Unknown	6 (11.8)

^a^ SD, standard deviation; ^b^ LDH, Lactate Dehydrogenase; ^c^ ECOG Eastern Cooperative Oncology Group; ^d^ GCB Germinal center B cell.

**Table 2:** Differences between early responders and late responders by D5PS.

Variable	Early Responder (*n* = 24)	Late Responder (*n* = 18)	*p*
Age			
Mean ± SD ^a^ (range)	55.4 ± 15.4 (30–81)	53.1 ± 14.1 (21–70)	0.614
≤60	14	11	0.856
>60	10	7	
Sex			
male	16	9	0.276
female	8	9	
Ann Arbor stage			
I–II	12	7	0.474
III–IV	12	11	
LDH ^b^			0.060 ^e^
normal	16	6	
elevated	8	12	
ECOG ^c^ Performance status			0.403
0–1	21	14	
2–4	3	4	
International Prognostic Index			0.641
0–1	11	5	
2	5	4	
3	5	5	
4–5	3	4	
Number of extranodal site			0.212
0–1	14	7	
≥2	10	11	
Subtype			0.393
GCB ^d^	8	8	
Non-GCB	13	9	
Not specified	3	1	

^a^ SD, standard deviation; ^b^ LDH, Lactate Dehydrogenase; ^c^ ECOG Eastern Cooperative Oncology Group; ^d^ GCB Germinal center B cell; ^e^ Fisher’s Exact Test.

**Table 3:** Changes of SULpeak and MTV compared to baseline PET/CT.

Compared PET Images	SULpeak		MTV	
	absΔSUL	%ΔSUL	absΔMTV (cm^3^)	%ΔMTV
**PET_∆0–1_**	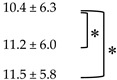	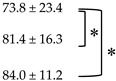	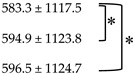	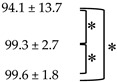
**PET_∆0–3_**				
**PET_∆0–6_**				

Values presented as mean ± SD. * denotes statistically significant difference with *p* value less than 0.05.

## Supplementary Materials

The following are available online at https://www.mdpi.com/2075-4418/10/12/1002/s1, Figure S1: %ΔSUL at each time point in detail for each patient; Table S1: %ΔSUL according to Deauville 5-point score; Table S2: The quantitative PET parameters at each time point according to disease status at 1 year; Table S3: Changes in SULpeak and MTV according to disease status at 1 year following therapy.
