# Supplementary material for: Early Interim Chemotherapy Response Evaluation by F-18 FDG PET/CT in Diffuse Large B Cell Lymphoma
Source: Diagnostics (Basel). 2020 Nov 24;10(12):1002. doi: 10.3390/diagnostics10121002 (PMC7761146; doi:10.3390/diagnostics10121002)
Supplement: Supplementary file 1 [file diagnostics-10-01002-s001.pdf]

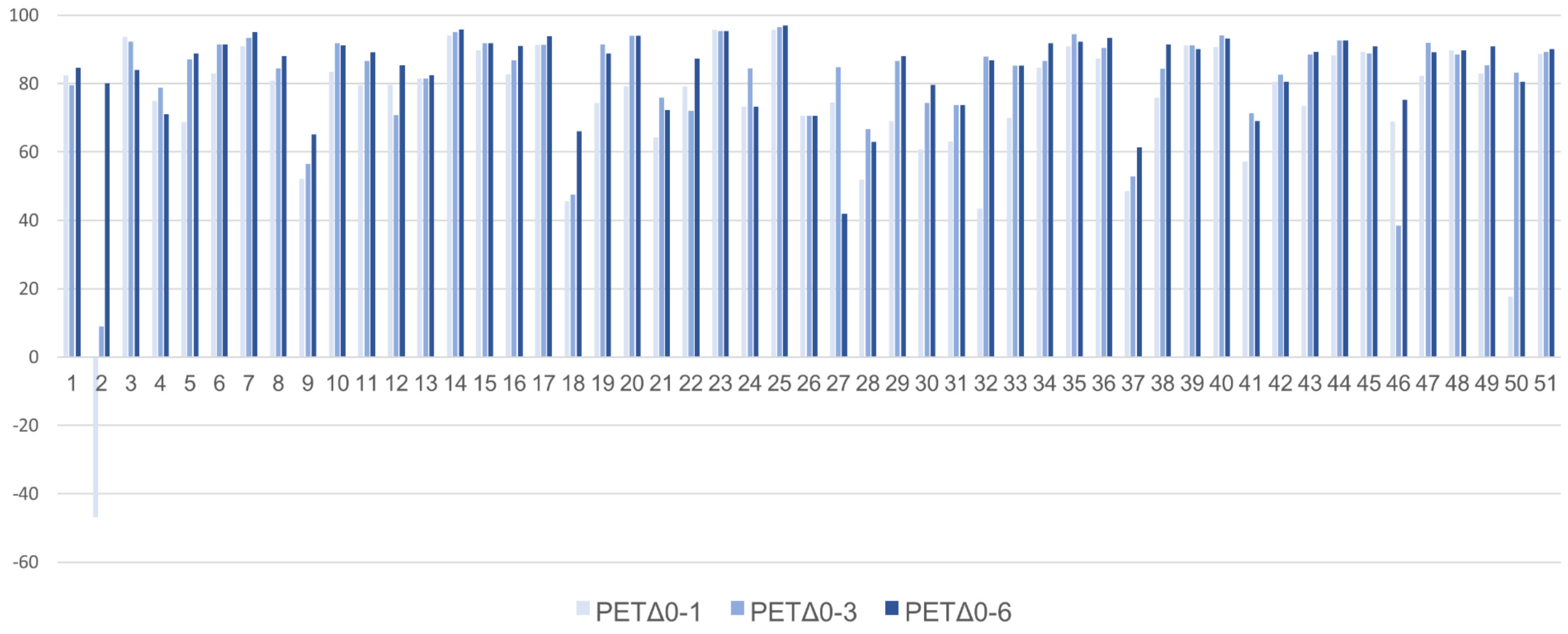

Figure S1. %ΔSUL in each time point in detail for each patient.

Table S1. %ΔSUL according to Deauville 5 point score.

Timing of PET		Deauville 5 point score				
		1	2	3	4	5
PET$_{\triangle 0\text{-}1}$	Mean ± SD<br>(range, n)	84.2 ± 10.7<br>(52.2~95.8, 18)	92.3 ± 2.0<br>(90.9~93.7, 2)	71.1 ± 16.3<br>(51.9~90.9, 6)	74.9 ± 12.0<br>(48.6~89.9, 12)	56.6 ± 36.4<br>(-46.9~83.4, 13)
PET$_{\triangle 0\text{-}3}$	Mean ± SD<br>(range, n)	85.2 ± 9.5<br>(56.5~96.6, 35)	91.7 ± 1.5<br>(90.4~93.4, 3)	78.9<br>(1)	80.3 ± 12.7<br>(52.9~92.3, 7)	50.5 ± 29.8<br>(9.0~85.4, 5)
PET$_{\triangle 0\text{-}6}$	Mean ± SD<br>(range, n)	86.2 ± 9.0<br>(63.0~97.0, 38)	87.6 ± 6.5<br>(80.5~93.4, 3)	91.0<br>(1)	78.0 ± 11.3<br>(61.4~87.3, 5)	66.8 ± 18.2<br>(41.8~84.1, 4)

Table S2. The quantitative PET parameters at each time point according to disease status at 1 year.

Parameters	Disease present (*n* = 9)	Disease-free (*n* = 42)	*P*
PET1 SULpeak	2.5 ± 1.8	3.0 ± 2.6	0.562
PET3 SULpeak	1.9 ± 1.6	2.1 ± 1.9	0.756
PET6 SULpeak	2.6 ± 3.2	1.7 ± 0.7	0.420
PET1 MTV (cm^3)	5.3 ± 8.6	15.8 ± 43.4	0.475
PET3 MTV (cm^3)	2.9 ± 7.7	2.2 ± 9.1	0.840
PET6 MTV (cm^3)	1.6 ± 3.2	0.6 ± 2.3	0.296

Table S3. Changes in SULpeak and MTV according to disease status at 1 year following therapy.

Parameters		Disease present (*n* = 9)	Disease-free (*n* = 42)	*P*
PET1	absΔSULpeak	10.4 ± 7.1	10.4 ± 6.2	0.976
	%ΔSULpeak	78.1 ± 15.3	72.8 ± 24.8	0.545
	absΔMTV(cm^3)	639.1 ± 667.3	571.4 ± 1198.0	0.871
	%ΔMTV	98.4 ± 1.9	93.2 ± 14.9	0.311
PET3	absΔSULpeak	11.0 ± 7.2	11.2 ± 5.8	0.911
	%ΔSULpeak	82.3 ± 15.3	81.2 ± 16.6	0.851
	absΔMTV(cm^3)	641.4 ± 668.2	585.0 ± 1205.1	0.893
	%ΔMTV	99.7 ± 0.6	99.2 ± 3.0	0.675
PET6	absΔSULpeak	10.3 ± 7.1	11.7 ± 5.5	0.522
	%ΔSULpeak	79.2 ± 18.2	85.1 ± 9.1	0.369
	absΔMTV(cm^3)	642.8 ± 669.2	586.6 ± 1206.1	0.893
	%ΔMTV	99.9 ± 0.2	99.6 ±1.9	0.645
